# Systemic Blood Immune Cell Populations as Biomarkers for the Outcome of Immune Checkpoint Inhibitor Therapies

**DOI:** 10.3390/ijms21072411

**Published:** 2020-03-31

**Authors:** Carlos Hernandez, Hugo Arasanz, Luisa Chocarro, Ana Bocanegra, Miren Zuazo, Gonzalo Fernandez-Hinojal, Ester Blanco, Ruth Vera, David Escors, Grazyna Kochan

**Affiliations:** 1 Oncoimmunology Group, Biomedical Research Centre Navarrabiomed-UPNA, IdISNA, Irunlarrea 3, 31008 Pamplona, Spain; carlos.hernandez.saez@navarra.es (C.H.); hugo.arasanz.esteban@navarra.es (H.A.); luisa.chocarro.deerauso@navarra.es (L.C.); ai.bocanegra.gondan@navarra.es (A.B.); miren.zuazo.ibarra@navarra.es (M.Z.); ester.blanco.palmeiro@navarra.es (E.B.); descorsm@navarra.es (D.E.); 2 Department of Oncology, Complejo Hospitalario de Navarra, IdISNA, Irunlarrea 3, 31008 Pamplona, Spain; gonzalo.fernandez.hinojal@navarra.es (G.F.-H.); ruth.vera.garcia@navarra.es (R.V.)

**Keywords:** immunotherapy, immune checkpoint inhibitors, systemic blood subsets, CD4^+^, CD8^+^, MDSCs

## Abstract

The development of cancer immunotherapy in the last decade has followed a vertiginous rhythm. Nowadays, immune checkpoint inhibitors (ICI) which include anti-CTLA4, anti-PD-1 and anti-PD-L1 antibodies are in clinical use for the treatment of numerous cancers. However, approximately only a third of the patients benefit from ICI therapies. Many efforts have been made for the identification of biomarkers allowing patient stratification into potential responders and progressors before the start of ICI therapies or for monitoring responses during treatment. While much attention is centered on biomarkers from the tumor microenvironment, in many cases biopsies are not available. The identification of systemic immune cell subsets that correlate with responses could provide promising biomarkers. Some of them have been reported to influence the response to ICI therapies, such as proliferation and activation status of CD8 and CD4 T cells, the expression of immune checkpoints in peripheral blood cells and the relative numbers of immunosuppressive cells such as regulatory T cells and myeloid-derived suppressor cells. In addition, the profile of soluble factors in plasma samples could be associated to response or tumor progression. Here we will review the cellular subsets associated to response or progression in different studies and discuss their accuracy in diagnosis.

## 1. Introduction

The roots of cancer immunology can be dated back to the nineteenth century. However, it is in the past few decades when immunotherapies have emerged as realistic options for cancer treatment. One of the pillars of this radical change has been the encouraging results obtained in several clinical trials with immune checkpoint inhibitors (ICI) such as anti-CTLA4, anti-PD-1 and anti-PD-L1 antibodies. Nowadays, the anti-CTLA4 ipilimumab (Bristol-Meyers Squibb), the anti-PD-1 nivolumab (Bristol-Meyers Squibb), pembrolizumab (Merck), and the anti-PD-L1 atezolizumab (Roche) are approved for the treatment of numerous cancers (e.g., melanoma, NSCLC, RCC).

However, despite of durable clinical benefits in many patients, a significant number of patients are still refractory ICI therapies. Hence, the early identification of potential responders and progressors would be a very important step forward. This is of special importance, as some of the treated patients develop hyperprogressive disease. Ideal biomarkers would enable the stratification of patients before the start of immunotherapies, and also the real-time monitoring of response for the duration of the treatments. 

One of the most extensively used biomarkers is tumor expression of PD-L1. There is ample evidence that PD-L1 overexpression correlates with immune evasion of immunogenic tumors. Therefore, the percentage of PD-L1 expression in tumor biopsies is used as a stratification factor for pembrolizumab administration in non-small cell lung cancer (NSCLC) or melanoma [1,2,3,4]. However, the usefulness of PD-L1 expression as a biomarker is still under debate, as patients with PD-L1-negative cancer cells can also respond to ICI therapies. Moreover, many PD-L1 detection protocols are not currently standardized [5]. For example, PD-L1 expression in myeloid cells may also play a role in response or resistance for the treatment of PD-L1 negative tumors, even when anti-PD-L1 antibody (atezolizumab) was the treatment of choice [6]. In these cases, the relative abundance of PD-L1^+^ CD11b^+^ myeloid cells in systemic blood correlated with clinical responses in lung cancer. These results indicated that PD-L1 expression in systemic immune cell populations can be a potential predictive biomarker of responses to PD-L1/PD-1 blockade therapy.

The tumor mutational burden (TMB) has recently gained popularity as a predictive biomarker associated with ICI responses. TMB provides a quantification of the number of mutations per megabase of genomic DNA within the tumor encoding genome. Indeed, there is a high mutational heterogeneity in different tumors types [7,8]. Patients with high TMB achieved a higher overall response rate (ORR) and a higher median progression-free survival (PFS), and therefore a better response to immunotherapy in NSCLC [9]. In addition to TMB, cancer DNA mismatch repair (MMR) gene mutations are quite relevant, and they are used as clinically applicable biomarkers [10,11]. It has been reported that MMR predicted clinical benefit of pembrolizumab in colon cancer [12]. Thus, tumor DNA-based sequencing approaches could be a key approach for the identification of patients who might respond to therapy.

The identification of biomarkers in peripheral blood that may accurately correlate with response to ICI therapies could provide a simple non-invasive method for patient selection, or even for treatment monitoring. The quantification of some of the biomarkers mentioned above requires tumor samples and biopsies that are not always available from every patient, or lacking at different time points during therapy. Biomarkers available before the start of ICI therapies can come from multiple sample types, and these can be useful for decision-making on specific treatment options. However, blood biomarkers can be a better option during the follow-up of patients along the duration of therapies. Blood samples provide two different data types: (1) Soluble biomarkers in plasma or serum, and (2) the composition of immune cell populations in whole blood or peripheral blood mononuclear cell (PBMC) fraction. Quantification of soluble biomarkers has been reviewed elsewhere [13,14]. In this review we will focus on immune cell populations in peripheral blood as predictors of responses to ICI therapies, and the immune-related mechanisms that ICI therapies activate through these cells.

## 2. Total Cell Counts and Ratios

Some of the immune correlates classically used to predict response or progression include changes in the lymphocyte/neutrophil compartment and blood cell counts with prognostic value [15,16,17]. For example, an absolute lymphocyte count (ALC) ≥ 1000/μL and neutrophil count (ANC) <4000/μL during treatment correlates with increased overall survival in melanoma patients treated with nivolumab [18,19]. It is not surprising that classical prognostic variables such as neutrophil-to-lymphocyte ratio (NLR) or serum lactate dehydrogenase (LDH) have been evaluated as potential predictors of response or resistance. Hence, high pretreatment NLR ratios (>2.2) and high LDH correlate with progression in nivolumab-treated patients with advanced NSCLC [20]. This is in agreement with other studies that utilize NLR as a biomarker in oncology [18,21,22].

## 3. CD8 T Cells

Cytotoxic CD8 T cells (cytotoxic T lymphocytes, CTLs) are considered the main effectors of anti-tumor immune responses. CTLs differentiate from naïve CD8 T cells activated by antigen-presenting cells (APCs) in lymphoid organs. In the context of anti-tumor immunity, cytotoxic T cells recognize tumor-derived peptides complexed to MHC molecules through binding of their T cell receptor (TCR) by interacting with APCs. Other sources of CTLs derive from activation of circulating central and effector memory T cells. This mechanism allows the rapid activation of a faster immune response after re-encounter with tumor antigens. After activation of naïve T cells or after differentiation from memory T cells, CTLs proliferate and migrate to the tumor, where these exert cytotoxic activities over cancer cells using different mechanisms. It is in the tumor microenvironment (TME), however, where CTLs can be inhibited by inhibitory signals delivered from cancer and immunosuppressive cells. 

The balance between CTL cytotoxicity and the immunomodulatory action of the TME leads to three possible scenarios: Tumor suppression, equilibrium, or tumor evasion. The importance of these interactions in the TME is exemplified by the expression of immune checkpoint inhibitor ligands such as PD-L1. And a high infiltration of T cells in the TME is on the other hand and in general terms a good indicative of ICI efficacy. 

Considering that T cell activation results in strong proliferation, it is not surprising that the number of total lymphocytes (assessed by parameters as absolute lymphocyte counts, or ALC) or specific T cell subsets (CD8 or CD4) might be considered good predictors of outcomes after ICI therapies. In a study with 82 melanoma patients treated with ipilimumab, Martens et al. found that an early increase in ALC 2 to 8 weeks after the start of treatments, followed by an increase in the relative percentages of CD4 and CD8 T cells (8-14 weeks after starting with treatment) correlated with improved survival [23]. Furthermore, the expression of proliferation markers such as Ki67 can also provide a more exact quantification of proliferation. A low baseline percentage of Ki67^+^ EOMES^+^ CD8 T cells in blood was associated with a higher possibility of relapse in melanoma patients treated with ipilimumab [24]. EOMES is a transcription factor that regulates expression of IFN-γ, granzyme B and perforin, being also a marker for T cell activation. The authors of this study showed that patients stratified according to a higher baseline proportion of Ki67^+^ EOMES^+^ CD8 T cells had an improved relapse-free survival. 

The expression of immune checkpoints in peripheral blood T cells has also been studied as a stratifying parameter as well, alone or in combination with proliferation markers. Jacquelot et al. recruited 190 metastatic melanoma patients treated with ipilimumab from 8 cohorts, and showed that high PD-L1 expression in CD8 T cells was associated with worse OS [25]. Kamphorst et al. also observed an increase of circulating HLA-DR^+^ CD38^+^ Ki67^+^ PD-1^+^ CD8 T cells in NSCLC patients following anti-PD-1 treatment which correlated with good responses. CD8 T cells in patients with a two-fold increase in the Ki67^+^ PD-1^+^ subset showed also increased CD28 expression, which implies that CD28^+^ CD8 T cells play a major role in objective responses to ICI therapies [26]. Another study expanded this observation by quantifying the ratio of Ki67^+^ PD1^+^ CD8 T cells after pembrolizumab treatment to baseline tumor burden as a good indicator of prolonged PFS in melanoma patients [27].

The distribution of memory *versus* non-memory cells in CD8 T cell populations has been associated to anti-CTLA4 therapy responses to but not to anti-PD-1 therapy in melanoma patients. High baseline percentages of effector memory CD8 T cells correlated with longer OS and with enhanced clinical responses [28,29,30]. However, a study in NSCLC patients receiving nivolumab uncovered that patients with a high central memory/effector CD8 T cell ratio had longer PFS [31].

## 4. CD4 T Cells

The recent past years have witnessed the surge of CD4 T cells into the scene of tumor immunity. Naïve CD4 T cells recognize tumor antigens similarly to CD8 T cells, but differing in the mode of presentation by APCs (MHC-II *versus* MHC-I). After activation, CD4 T cells proliferate and differentiate into helper subsets (Th1, Th2, Th9, and Th17) or regulatory T (Treg) cells depending on the cytokines and other factors present during their differentiation. Some of these CD4 T cells possess anti-tumor activities, while others exert immunosuppressive activities mainly by regulating the CD8 response.

Overall, most studies have broadly found equivalent changes in CD8 and CD4 T cells during anti-tumor responses. Thus, strong CD4 proliferation has been associated with good prognosis in agreement with CD8 responses [23,24]. In contrast, high expression of immune checkpoints both in CD4 and CD8 T cells correlates with resistance to therapy [25].

According to the identification of specific CD4 T cell subsets, a study including 46 metastatic melanoma patients treated with nivolumab showed that increase in Th9 frequency in responders, which also correlated with higher levels of serum TGFβ and higher percentages of IL4-producing CD4 T cells [32]. The authors of this study proposed that Th9 cells possessed anti-tumor capacities by regulating the expression of cytotoxic molecules by CTLs. 

We have been interested for several years in PD-L1/PD-1 signaling mechanisms in the context of antitumor immunity. We carried out a recent translational project quantifying the relative percentages in peripheral blood of CD4 and CD8 T cell differentiation subsets in NSCLC patients treated with anti-PD-1/PD-L1 immunotherapies [33]. T cells can be classified according to CD27 and CD28 expression profiles into poorly differentiated (CD27^+^CD28^+^), intermediately differentiated (CD27^-^CD28^+^), and highly differentiated (CD27^-^CD28^-^) subsets. Patients were stratified into two groups by an approximately baseline cut-off value of 40% CD27^-^ CD28^-^ highly differentiated CD4 T cells. Interestingly, objective responders had percentages above this cutoff value, while patients with a percentage below this cut-off were refractory to the treatment. Hence, patients with a high percentage of highly differentiated CD4 T cells showed longer PFS and OS. Interestingly, no clear correlation was found between the relative percentages of baseline CD8 T subsets with the efficacy of immunotherapies. Moreover, highly differentiated CD4 T cells corresponded to both central and effector memory cells but not to senescent or exhausted cells. Our results were also in very close agreement by a detailed and complete study carried out by Kagamu et al. These authors used mass cytometry and found that NSCLC patients responding to nivolumab had a significantly higher percentage of CD62L^low^ CD4 T cells than non-responders at baseline [34]. Interestingly, these T cells were also double negative in CD27 and CD28, and corresponded to memory subsets. Importantly, the cut-off values from our study and their study were found to be nearly the same, strongly suggesting that CD4 T cell quantification in peripheral blood is a predictive biomarker with clinical value. Moreover, in a recent study including NSCLC and RCC patients treated with nivolumab and pembrolizumab, authors also highlighted the relevance of central memory CD4 T cells for tumor immunity. The baseline percentage of central memory CD4 T cells was higher in responder patients or patients with stable disease than in patients with progressive disease [35]. Taken together, the data from these independent studies highlight the relevance of the CD4 systemic immunity for anti-tumor immunity and clinical responses to ICI therapies.

Tregs constitute a special immunosuppressive subset that can be differentiated in the thymus (natural Tregs) or from naïve CD4 T cells (inducible Tregs). Tregs promote immunosuppression and tolerance once infiltrated into the TME. Accordingly, a decrease in peripheral blood Tregs after ipilimumab treatment in metastatic melanoma patients was associated to disease control and OS [36], while the baseline percentage of CD25^+^ FoxP3^+^ CD4 T cells in NSCLC patients treated with nivolumab was higher in non-responders [34]. These results overall suggest that a decrease of this immunosuppressive population contributes to the efficacy of ICI therapies. However, another study found that baseline percentages ≥ 1.5% of CD4^+^ CD25^+^ FoxP3^+^ Tregs were associated with good prognosis in patients receiving ipilimumab [37]. In agreement with this latter study, Tarhini et al. showed that an increase in Treg percentage in advanced melanoma patients treated with neoadjuvant ipilimumab correlated with prolonged PFS [38]. A potential explanation for these discrepancies may lay on the varying suppressive capacities of Tregs following ICI therapy. For example, Woods et al. found that metastatic melanoma patients responding to nivolumab showed an increase in circulating Tregs, but these cells had increased phosphorylated STAT3 which correlated with reduced immunosuppressive activity [39].

## 5. NK Cells

Natural killer (NK) cells group several types of lymphocytes with an innate capacity to recognize and eliminate tumor cells, without the need of a previous immunization to become activated. There are few studies on circulating NK cells as potential biomarkers of responses to ICI therapy, probably because their activities are mainly exerted within the TME [40].

In a study using CyTOF mass cytometry, Subrahmanyam et al. found that melanoma patients responding to anti-PD-1 antibodies had a baseline higher expression of CD69 and MIP-1β in NK cells stimulated with PMA and ionomycin [28]. This study indicates a potential role for differentially activated NK cells in the response to ICI therapy, as unstimulated cells showed comparable CD69 and MIP- between responders and non-responders.

## 6. MDSCs

Myeloid-derived suppressor cells (MDSCs) constitute a heterogeneous group of myeloid cells with immunosuppressive activities that expand particularly during in chronic inflammatory conditions such as cancer [41,42]. MDSCs are usually divided in two major subsets: Monocytic (mMDSCs) and granulocytic MDSCs (gMDSCs or polymorphonuclear (PMN)-MDSCs). The increase in circulating MDSCs is a consequence of an altered myelopoiesis and generally correlates with MDSC infiltration in the TME. Many reports have shown the relationship between the number of MDSCs and treatment outcome in cancer patients. Indeed, the percentage of peripheral blood MDSCs has some prognostic value for ICI therapy. 

Sade-Feldman et al. showed that the baseline percentage of circulating total CD33^+^ CD11b^+^ HLA- DR^-^ MDSCs in melanoma patients treated with ipilimumab inversely correlated with response and OS [43]. However, the authors did not discriminate between mMDSCs and gMDSCs. Furthermore, several studies have found that melanoma patients with lower baseline numbers of circulating mMDSCs had a higher chance of responding to ipilimumab treatment with improved OS [37,44,45,46]. This correlation is not restricted to ICI immunotherapies, as it was also found in castration-resistant prostate cancer patients treated with the cancer vaccine GVAX plus ipilimumab [47]. In addition, other studies in melanoma patients showed the relevance of changes in mMDSC relative numbers following treatment with ipilimumab. Coaña et al. showed that patients with clinical benefit had a significant reduction in mMDSC percentages right after the first dose of ipilimumab, even though no differences were found in baseline percentages between responders and non-responders [48]. This study is in agreement with others demonstrating that patients with a decrease in mMDSCs after the first doses of ipilimumab had increased survival [38,49]. Interestingly, Kitano et al. showed that the percentage of mMDSCs was inversely correlated with increase CD8 T cell absolute numbers following ipilimumab treatment [50]. Taken together, these studies suggest that low baseline percentages of peripheral mMDSCs before starting ICI therapy, or their decrease after the start of therapy are indicators of positive outcomes. 

## 7. Monocytes and Macrophages

Tumor-associated macrophages (TAMs) are classically one of the main immunosuppressive myeloid cells infiltrating the TME. TAMs can directly derive within the TME from resident macrophages through polarization towards a M2-like phenotype or from monocytes and mMDSCs through differentiation. However, most TAMs arise from the circulating pool of classical “uncommitted” monocytes. It has to be taken into account that tumor infiltration with monocyte-derived M1-polarized macrophages does have anti-tumor activities in the TME. In blood, monocytes are classified according to CD14 and CD16 expression profiles. CD14^high^CD16^-^ correspond to classical monocytes which are recruited to tissues following inflammation or damage; CD14^high^CD16^dim^ consists of intermediate monocytes; and CD14^low^CD16^high^ correspond to non-classical monocytes with surveillance functions. Nevertheless, the abundances of distinct circulating monocytes are not reflecting what takes place in the tissue, and there are only a few indications about changes in blood monocytes associated with clinical outcome after ICI therapy.

A study in melanoma patients treated with ipilimumab uncovered that responders displayed higher baseline percentages of non-classical monocytes than non-responders [51]. Moreover, an increased frequency of baseline CD14^+^ CD16^-^ HLA- DR^high^ classical monocytes as quantified by CyTOF correlated with enhanced PFS and OS in melanoma patients treated with anti-PD-1 therapy [17]. Further research will be needed to understand the participation of each subset of circulating monocytes in tumor immunity, and their utility as predictors of therapeutic efficacy. 

## 8. Immune Cell Populations in Patients with Hyperprogression after ICI Therapy 

A series of independent research teams have reported a deleterious effect of PD-1/PD-L1 blockade immunotherapy, termed hyperprogressive disease (HPD). HPD is characterized by an acceleration of tumor growth kinetics resulting in fast progression associated to quick clinical deterioration, although the specific clinical criteria for its definition are still controversial [52,53,54]. The mechanisms of HPD are largely unknown, although two recent studies have proposed as possible causes the binding of anti-PD-1 IgG4 with the Fc receptor in macrophages, or proliferation of tumor-infiltrating PD-1 expressing Tregs [55,56]. The identification of reliable biomarkers for detection of patients with a high probability of developing HPD after ICI therapies is a matter of special importance. 

Several clinical factors have been linked to HPD, including age ≥ 65 years [52], liver metastases [54] or locoregional relapse in head and neck squamous cell cancer (HNSCC) [53] among others. According to the identification of predictive biomarkers in peripheral blood, a retrospective study that included 62 advanced gastric cancer (AGC) patients treated with nivolumab found an association of several biomarkers to HPD. Levels of serum C-reactive protein (CRP) (median 4.0 *versus* 0.50 mg/dL, *p* = 0.006), lactate dehydrogenase (LDH) (median 396.0 *versus* 179.5 U/L, *p* = 0.006) or absolute neutrophil count (ANC) (median 4490 *versus* 2720/μL, *p* = 0.002) were significantly higher in patients that ended up developing HPD. Furthermore, ANC and CRP remained elevated 4 weeks after the start of immunotherapy with nivolumab in patients with HPD (7740 *versus* 4490/μL and 8.3 *versus* 4.0 mg/dL respectively) [57]. No prospective studies of any biomarker associated to HPD have been published so far with the exception of one [58]. In the study by Arasanz et al., peripheral blood immune cell populations were quantified by flow cytometry in patients with advanced NSCLC treated with immune-checkpoint inhibitors (ICI) as second or further lines of therapy. A sharp expansion of highly differentiated CD4 T cells between the first and the second cycle of immunotherapy was observed in HPD patients. An increase of this population superior to 30% identified HPD patients with a 82% specificity and 70% sensitivity. Tumor growth ratio (TGR), as defined by Champiat et al. [54] was significantly higher for HPD patients compared to non-HPD progressors (2.67 *versus* 1.03, *p* = 0.0126). By multivariate analysis, only the expansion of highly differentiated CD4 T cells and PD-L1 tumor expression higher than 5% were the only independent factors associated with HPD.

Finally, a recent meta-analysis that included 9 retrospective studies has been published. This study confirmed the prognostic value of elevated serum LDH above the upper limit of normal (ULN), with an odds ratio (OR) of 1.89 (95% CI 1.34 to 2.57, *p* = 0.043), but without finding any significant value for the other peripheral blood variables under study [59].

## 9. Future Perspectives

We have reviewed here the current knowledge on the applicability of quantification of peripheral blood immune cell populations as biomarkers to assess the efficacy of ICI therapies. Nevertheless, cancer treatment is right now a continuously-changing field, and especially after the development of clinically effective immunotherapies. The approval of novel antibodies directed towards immune checkpoints other than PD-L1, PD-1, or LAG-3 together with the inclusion of combination therapies, is prioritizing the search for biomarkers of response to immunotherapies.

The improvement of well-established techniques such as flow cytometry, and the clinical application of novel high throughput technologies such as mass cytometry-CYTOF is allowing the identification and quantification of multiple cell subsets from small sample quantities. Accordingly, early biomarkers based on crude quantification of ALC and total CD4/CD8 T cells have moved towards the quantification of highly specific cell subsets. For example, highly-differentiated memory T cell subsets, proliferating Ki67^+^ PD1^+^ CD8 T cells or CD14^+^ CD16^-^ HLA-DR^high^ classical monocytes. More sophisticated technical approaches coupled to highly detailed multidimensional statistical analyses will expand our knowledge on the participation of peripheral blood cell subsets in the efficacy of cancer therapies. Although the introduction of some of these techniques in clinical practice can be complicated right now, perhaps our efforts should be driven towards the design of simpler analyses once the specific targets have been identified.

The expression of immune checkpoints in peripheral immune cells provides another level of information, although their quantification could result in a double-edged sword. Increased levels of immune checkpoints may identify T cells that could be re-activated with the use of the appropriate inhibitors. However, the up-regulation of alternative inhibitory molecules can indeed counteract the action of a particular inhibitor. In NSCLC patients under anti-PD-1/anti-PD-L1 immunotherapy that we have analyzed, most non-responders had T cells that simultaneous co-expressed PD-1 and LAG-3. Indeed, co-blockade with a combination of anti-PD-1 and anti-LAG-3 antibodies was sufficient to increase T cell activation from non-responder patients [33]. Fine mapping of the expression profiles of immune checkpoints in peripheral T cells could be indicative of the particular inhibitor combination to be used in these patients, especially when a plethora of other ICI are currently under study in different clinical trials. 

The expression of ligands for immune checkpoints in peripheral blood immune cells can also be considered to evaluate responses to ICI therapies. Jacquelot et al. found that PD-L1 expression in circulating CD8 T cells was associated with good prognosis in melanoma patients treated with ipilimumab [25] Although the expression of PD-L1 is mainly quantified in tumor cells or in infiltrating immune cells within the TME, it can also be highly expressed by peripheral blood myeloid cells. In a clinical case study, two tumor PD-L1-negative NSCLC patients with similar clinical history exhibited a remarkably different response to atezolizumab. The responder patients showed a high baseline percentage of PD-L1-expressing myeloid cells [6]. This observation was corroborated in an exploratory follow-up study of 31 patients with advanced NSCLC, suggesting that quantification of systemic PD-L1^+^ myeloid cell subsets could complement other biomarkers for patient stratification, independently of PD-L1 expression in tumor biopsies.

An emerging and promising candidate as a biomarker for immunotherapies is the quantification of the TCR repertoire and its changes in peripheral T cells before and during therapy. It was speculated that having a high TCR clonal diversity could correlate with higher probabilities to establish efficacious anti-tumor immune responses. Consequently, several studies have indeed associated a better response to a more diverse TCR repertoire in peripheral T cells [60,61,62,63].

## 10. Concluding Remarks

The search for specific biomarkers that predict the outcome of cancer patients after ICI therapy is still ongoing. Quantification of baseline immune cell populations in peripheral blood or during treatments is supporting the hypothesis that active CD4 and CD8 T cell proliferation and activation, high frequency of memory cells and low numbers of immunosuppresive cells are indicative of good prognosis (Table 1). These results could be added to other clinical data, including analyses of tumor biopsies if available for biomarkers such as TMB or PD-L1 expression (which is so far the only biomarker used in clinical settings).

**Figure 1:**
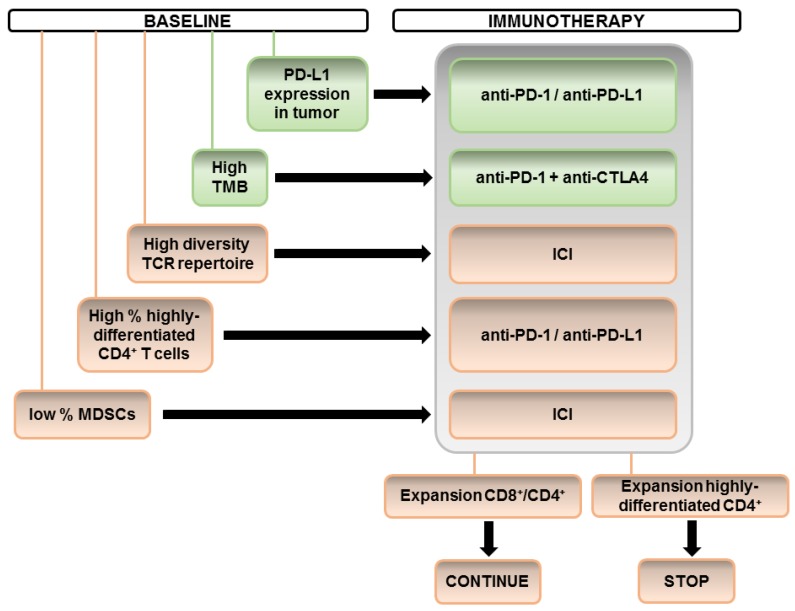
Suggested application of a sequential algorithm to patient selection for immune checkpoint inhibitors (ICI) therapy. Before starting ICI therapies, PD-L1 tumor expression and tumor mutational burden (TMB) determination can be provided as biomarkers under current clinical practice (green). Further integration of novel biomarkers for patient selection is presented in orange both for baseline variables or after the first cycle of treatment.

However, it is intriguing that we can find many apparently contradictory reports when comparing different studies. For example, studies that have found changes in CD8-based immunity sometimes do not simultaneously identify changes in CD4-dependent systemic immunity. A possible explanation could be the specific set of markers used by different studies and the variability in ICI treatments and patient cohorts. We propose the derivation of a sequential algorithm integrating the different biomarkers, taking into account our current understanding as shown in Figure 1.

Finally, special care must be taken to apply ICI therapy to patients with a high probability of developing HPD. For these patients, particular care should be taken. Biomarkers in this regard can help take the appropriate clinical decisions. 

## Figures and Tables

**Table 1:** Levels of specific peripheral immune cells correlated with clinical outcome of cancer patients under ICI therapy.

Cell Type	Correlation with Clinical Benefit or Prolonged OS/PFS	References
**CD8 T cells**	High proliferation	[24,37]
	High expression of immune checkpoints (PD-1)	[16,27]
	High memory CD8 T cell numbers	[28,29,30]
**CD4T cells**	Increased proliferation	[24,37]
	High frequency of Th9 cells	[32]
	High percentage of highly differentiated (CD27^-^CD28^-^) CD4 T cells	[33]
	High percentage of memory CD4 T cells	[33,34,35]
	Low Treg numbers or high numbers with reduced immunosuppressive activity	[23,36,39]
	High Treg percentage	[38]
**MDSCs**	Low baseline MDSC numbers	[23,44,45,46,47]
	Decreased MDSC numbers after ICI therapy	[38,48,49]
**Monocytes**	Increased baseline frequency of classical CD14^+^CD16^-^ monocytes	[17]

## Abbreviations

ALC	absolute lymphocyte count
ANC	absolute neutrophil count
APC	antigen-presenting cell
CTL	cytotoxic T lymphocyte
HPD	hyperprogressive disease.
ICI	immune checkpoint inhibitors
MDSC	myeloid-derived suppressor cell
NLR	neutrophil-to-lymphocyte ratio
NSCLC	non-small cell lung cancer
ORR	overall response rate
OS	overall survival
PBMCs	peripheral blood mononuclear cells
PFS	progression-free survival
RCC	renal cell carcinoma
TAM	tumor-associated macrophage
TMB	tumor mutational burden
TME	tumor microenvironment
Treg	regulatory T cell
